# Diagnosis of an atypical presentation of basal cell carcinoma of the nasal pyramid: Case report

**DOI:** 10.1016/j.ijscr.2024.109674

**Published:** 2024-04-20

**Authors:** B. Saout Arrih, W. Bijou, Y. Oukessou, S. Rouadi, R. Abada, M. Mahtar

**Affiliations:** Department of Otolaryngology, Head and Neck Surgery, Ibn Rochd University Hospital, Faculty of Medicine and Pharmacy, Hassan II University Casablanca, Morocco

**Keywords:** Case report, Basal cell carcinoma, Infiltrant, Diagnosis, Management

## Abstract

**Introduction and importance:**

Basal cell carcinoma is a common form of skin cancer whose global incidence is rising rapidly, with over 70 % of locations on the face. In contrast to their low mortality, their morbidity is high. Extensive basal cell carcinomas and infiltrative lesions are associated with a high recurrence rate, which can result a serious esthetic and functional damage.

**Case presentation:**

We report the case of a 65-year-old female patient, who consulted our ENT department for a large ulcerating lesion of the nasal pyramid. CT scan revealed a lesion of the nasal pyramid measuring 38 mm in long axis, which appeared to come into contact with the anterior part of the nasal septum. The pathological findings were consistent with an infiltrating basal cell carcinoma. The patient underwent surgical resection with reconstruction using a forehead flap.

**Clinical discussion:**

Following ANAES guidelines, when the diagnosis of a poor-prognosis BCC is uncertain, or when major reconstruction is required at the time of surgery, biopsy is strongly recommended to confirm the diagnosis. The evolution of BCCs is essentially local, and they rarely metastasize, with a maximum incidence rate of 0.55 %, of which around 85 % appears on the face. Thus, local extension of BCCs mainly involves adjacent tissues, including the perichondrium, in which case imaging is necessary to assess the extent of damage. The most common and effective treatment is surgical excision, with a margin of healthy tissue around the tumor.

**Conclusion:**

Because early diagnosis and carcinological excision are the keys to a good prognosis. We must insist on the role of primary and secondary prevention, and on the importance of early diagnosis.

## Highlights

• The nasal pyramid is a highly affected area by basal cell carcinoma.
• Infiltrating basal cell carcinomas have a greater sub-clinical extension than superficial and nodular forms.
• Lesions of the nasal pyramid are complex to remove and in reconstruction, especially when the tumor is large.
• Frontal flap is the most used flap.
• We insist on the role of primary and secondary prevention, and the importance of early diagnosis at a stage when treatment is codified.

## 1 Introduction

Basal cell carcinomas are the most common skin cancers worldwide. Their prevalence has been rising in recent decades due to longer life expectancy and changing behavioral habits, particularly prolonged and intense exposure to UV radiation [1,2].

The nasal pyramid gives the face a large part of its character and is involved in social interaction. As a result, tumors of this area can cause loss of tissue usually superficial but sometimes transfixing, requiring not only complete surgical excision but also appropriate reconstruction methods.

Although surgery remains the foundation stone of treatment, it is still subject to the constraints of mutilation in advanced cases, despite the advances reported in reconstructive surgery.

Based on these data, we report the case of a locally advanced infiltrating basal cell carcinoma of the nasal pyramid.

This case report has been reported in line with the SCARE criteria [10].

## 2 Case report

We report the case of a 65-year-old female patient, currently receiving medication for high blood pressure, who consulted our ENT department for an ulcerating lesion of the nasal pyramid.

The history of the disease dates to one year ago, with the appearance of this highly bleeding lesion, without any other associated ENT symptoms, in particular no cervical lymph nodes.

**Fig. 1:**
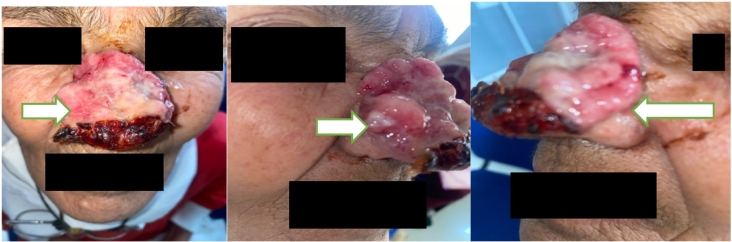
Picture showing the ulcerating lesion covering the entire nasal pyramid.

On physical examination, the patient was in good general condition, with a clean, moist tongue. On rhinological inspection, we note an ulcerative lesion covering the entire nasal pyramid and stopping at the peri-orifice region of the nose (Fig. 1).

**Fig. 2:**
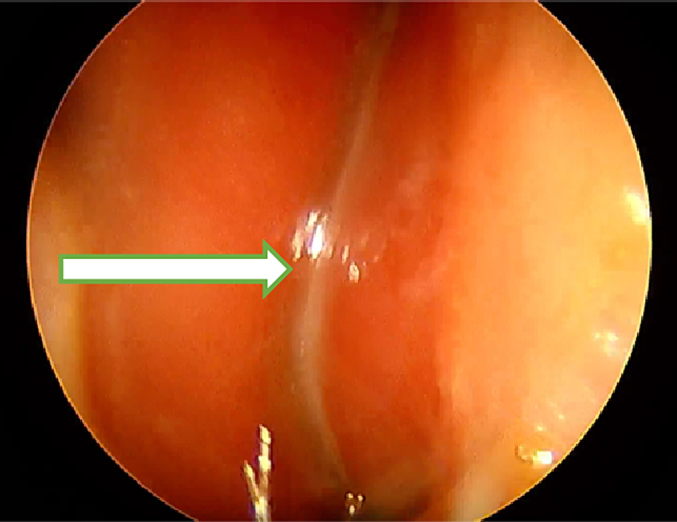
Image showing wide septal deviation of the left nasal fossa and inferior turbinate hypertrophy with turbino-septal junction.

**Fig. 3:**
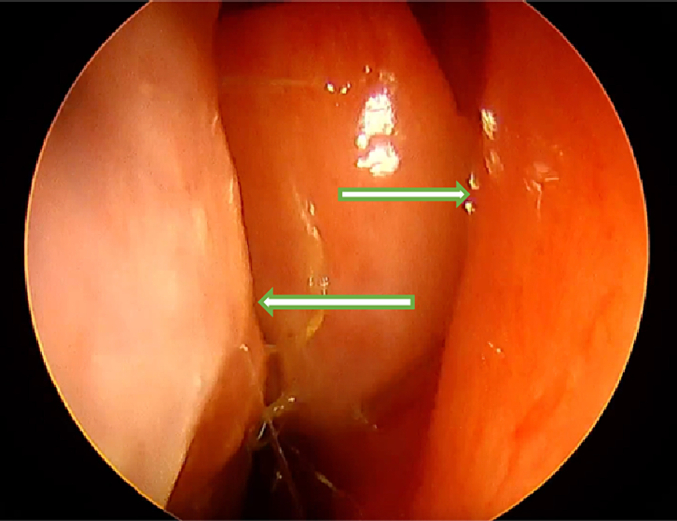
Rhinoscopy of the right nasal fossa: septal deviation with intact lower and middle meatus.

On rhinoscopy, the left nasal cavity showed a wide septal deviation and inferior turbinate hypertrophy with turbino-septal junction, preventing exploration of the rest of the nasal cavity (Fig. 2). In the right nasal cavity, septal deviation is also noted, but the inferior and middle meatus are unobstructed (Fig. 3). The rest of the physical examination was normal.

**Fig. 4:**
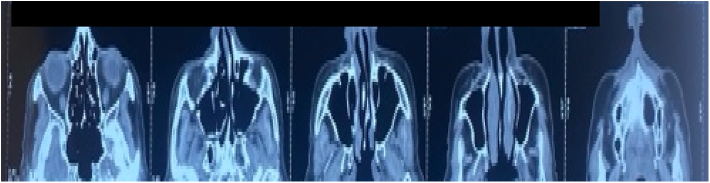
CT scan image showing the location of the process which comes into contact with the anterior part of the nasal septum (white arrow), without extension to the nasal cavity (orange arrow).

**Fig. 5:**
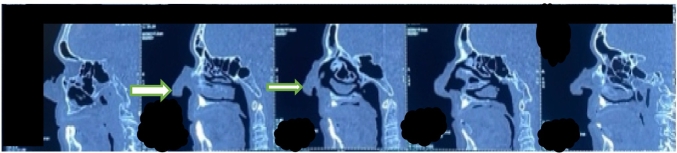
Picture showing the CT scan aspect of the ulcerative process of the nasal pyramid.

As part of the paraclinical examination, a CT scan of the face revealed an ulcerated, budding lesion of the nasal pyramid measuring 38 mm in long axis, which appeared to come into contact with the anterior part of the nasal septum, with no clearly detectable extension to the nasal cavities (Fig. 4, Fig. 5).

A biopsy of this process was performed, showing a pathological result consistent with an infiltrating basal cell carcinoma (Fig. 7).

On the basis of these data, the decision was made to perform a surgical resection after preparing the patient by starting a drug treatment combining antibiotics for 10 days and corticosteroids for 5 days.

Surgery was performed by an ENT specialist in two surgical procedures.

**Fig. 6:**
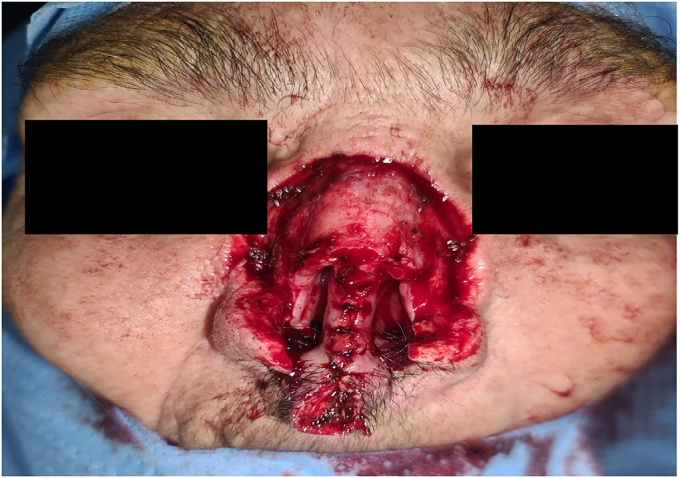
Image showing the result of total surgical excision of the tumor.

The 1st stage consisted of total surgical removal of the tumor, with a 6-10 mm limit of removal including the anterior part of the septum (Fig. 6). The postoperative evolution was excellent, showing positive outcome after 3 months with no tumor recurrence.

**Fig. 7:**
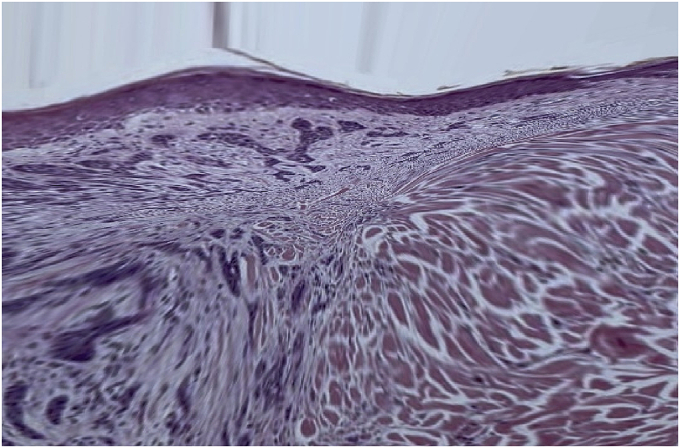
Histological image showing the result of pathological examination.

The 2nd stage of surgery consists of reconstruction of the nasal pyramid using a frontal flap.

Reconstruction with a forehead flap is scheduled one year after tumor resection surgery. The reason for delaying the 2nd stage surgery is the large size of the tumor, which is not always easy to remove with good safety margins. Reconstruction will therefore be performed 1 year after the primary surgery, making sure that there is no recurrence.

The postoperative evolution was excellent, showing positive outcome after 3 months with no tumor recurrence.

## 3 Discussion

Malignant tumors of the nasal pyramid are essentially represented by carcinomas. Basal cell carcinoma is the most common type, accounting for 61 % of skin cancers, followed by squamous cell carcinoma, whose incidence is rising rapidly [1].

G. Staub has reported that there is no difference in incidence between genders [3].

The most commonly reported causal factor is UV exposure. It is known to damage the genome of epithelial cells, either directly through radiation and/or indirectly through the generation of free radicals [2].

In contrast to squamous cell carcinoma, basal cell carcinoma may not be associated with a precancerous lesion [4].

The nasal pyramid is a highly affected area, as evidenced by numerous studies. Case series by “*Compte*” found predominance of nasal wings in 51 % [5].

There is a predominance of the burgeoning-ulcer aspect of the lesion, followed by the ulcerative aspect. Microscopic presentation shows proliferation in the form of massive or trabecular cell aggregates, made up of small basophilic cells. Infiltrating basal cell carcinomas have a greater sub-clinical extension than superficial and nodular forms, which is why they are likely to be subject to incomplete excision. The National Agency for Health Accreditation and Evaluation recommends an excision margin of 3 mm to 10 mm [6,7].

In most cases, it is impractical to establish an indication on clinical evidence only. Complementary workup is therefore decisive. The use of imagery helps to clarify the surgical approach. Thanks to its excellent diagnostic sensitivity in the soft tissues, MRI is the key examination for identifying, assessing and monitoring the local evolution of the tumor. In cases where subjacent bone extension is suspected, it is recommended to perform a CT scan with bone reconstruction [8].

Surgical excision is the first option in treating tumors of the nasal pyramid, other therapeutic modalities such as cryotherapy, chemotherapy and rarely radiotherapy may be indicated [9].

Skin tumors are the most common in the elderly. For this reason, they are often difficult to manage. Lesions of the nasal pyramid are particularly complex to remove. The incision is drawn before any anesthetic infiltration, which is performed at the periphery of the tumor without infiltrating it, to avoid tumor dissemination. The resection technique must be impeccable, respecting safety margins as far as possible [10].

There are many ways of repairing the tissue loss created by excision of these tumors, and the choice of repair procedure is sometimes difficult. Total skin grafts are described in which the entire thickness of the skin is removed. The donor area, which could only scar from its edges, must be sutured. These total skin grafts give the best results [11].

Surgical resection of large, infiltrating basal cell carcinomas of the nasal pyramid produces extensive tissue loss, requiring flap repair.

The upper part of the nose concerns the bony nose, where loss of substance is purely cutaneous, and consists of the dorsum and lateral surfaces. Several types of flaps can be used depending on the size of the loss of substance: if it is less than 2 cm, a Rieger flap can be used; if it exceeds 2 cm, a median frontal flap or an oblique frontal flap is preferred [12].

The lower part of the nose involves the cartilaginous nose, and loss of substance can be transfixing, necessitating a multi-layer reconstruction with a risk of excess thickness. The lower part of the nose is made up of three parts: the nasal wing, the tip and the columella. Often the choice is to perform a medial frontal flap with a cartilage graft if the cartilage is injured [13].

Large losses of tissue of the nose are delicate to reconstruct. In most cases, the loss of substance is transfixing in the lower part, requiring a multi-layer reconstruction with repair of the mucosal plane using septal mucosa flaps, repair of the cartilaginous plane with reinforcement of the free edge of the nostril using concha cartilage, and repair of the cutaneous plane using a frontal flap [14].

Basal cell carcinomas have an excellent prognosis in the vast majority of cases, when the surgical management is radical. Metastasis of basal cell carcinomas is exceptional, although cases have been described in the literature (estimated at 0.02 % to 0.5 %). Recurrences do exist, and may be related to the type of carcinoma, the affected site and the type of treatment, particularly when it is non-surgical. When aggressive, as in the scleroderma type, invasion can destroy the orbital and nasal cavities [15].

All patients with basal cell carcinoma should be monitored to detect recurrence or new lesions as early as possible. For aggressive or infiltrative lesions, follow-up every 6 months is recommended, and for others, every year. Photoprotection measures, which are always necessary for these patients, should not be forgotten [16,17].

## 4 Conclusion

Basal cell carcinomas of the nasal pyramid are a skin cancer encountered in the daily practice of ENT surgeons. Treatment is essentially surgical in accordance with carcinological guidelines, repair of tissue loss using a range of different procedures provides acceptable functional and esthetic results. We must insist on the role of primary and secondary prevention, and on the importance of early diagnosis at a stage when treatment is simple and codified. Because early diagnosis and carcinological excision are the keys to a good prognosis.

Education is needed to make patients aware of the harmful effects of UV exposure.

## Provenance and peer review

Not commissioned, externally peer-reviewed.

## Ethical approval

Ethical approval was provided by the authors institution.

Written informed consent was obtained from the patient for publication of this case report and accompanying images. A copy of the written consent is available for review by the Editor-in-Chief of this journal on request.

## Sources of funding

None.

## Research registration

N/A.

## Declaration of competing interest

None.
